# Ten Years of Pediatric Lung Ultrasound: A Narrative Review

**DOI:** 10.3389/fphys.2021.721951

**Published:** 2022-01-06

**Authors:** Anna Maria Musolino, Paolo Tomà, Cristina De Rose, Eugenio Pitaro, Elena Boccuzzi, Rita De Santis, Rosa Morello, Maria Chiara Supino, Alberto Villani, Piero Valentini, Danilo Buonsenso

**Affiliations:** ^1^Pediatric Emergency Unit, Department of Emergency and General Pediatrics, Bambino Gesù Children’s Hospital, IRCCS, Rome, Italy; ^2^Department of Imaging, Bambino Gesù Children’s Hospital, IRCCS, Rome, Italy; ^3^Department of Woman and Child Health and Public Health, Fondazione Policlinico Universitario A. Gemelli IRCCS, Rome, Italy; ^4^General Pediatric and Infectious Disease Unit, Internal Care Department, Bambino Gesù Children’s Hospital, Rome, Italy; ^5^Dipartimento di Scienze Biotecnologiche di Base, Cliniche Intensivologiche e Perioperatorie, Università Cattolica del Sacro Cuore, Rome, Italy; ^6^Global Health Research Institute, Istituto di Igiene, Università Cattolica del Sacro Cuore, Rome, Italy

**Keywords:** lung ultrasound, LUS, children, pediatrics, imaging, lung disease

## Abstract

Lung diseases are the most common conditions in newborns, infants, and children and are also the primary cause of death in children younger than 5 years old. Traditionally, the lung was not thought to be a target for an ultrasound due to its inability to penetrate the gas-filled anatomical structures. With the deepening of knowledge on ultrasound in recent years, it is now known that the affected lung produces ultrasound artifacts resulting from the abnormal tissue/gas/tissue interface when ultrasound sound waves penetrate lung tissue. Over the years, the application of lung ultrasound (LUS) has changed and its main indications in the pediatric population have expanded. This review analyzed the studies on lung ultrasound in pediatrics, published from 2010 to 2020, with the aim of highlighting the usefulness of LUS in pediatrics. It also described the normal and abnormal appearances of the pediatric lung on ultrasound as well as the benefits, limitations, and possible future challenges of this modality.

## Introduction

Lung diseases are the most common conditions in newborns, infants, and children and are also the primary causes of death in children younger than 5 years old (Liu et al., 2015b). Therefore, accurate and timely diagnosis is extremely important in order to enable efficient treatment and to improve the prognosis of patients with lung diseases.

In the past, the diagnosis of lung disease in the pediatric population mainly depended on chest X-ray (CXR) and/or computed tomography (CT). However, CXR may not be efficient bedside, requires transfer to specific radiological settings with all the infectious risks for patients and operators, and exposes the patient to ionizing radiation. Ultrasound imaging is based on the reflection and scattering of ultrasound (US) beam occurring at the interfaces between different media.

Traditionally, the lung was not thought to be a target for ultrasound waves due to their inability to penetrate the gas-filled anatomical structures. With the deepening of knowledge on ultrasound in recent years, it is now known that the affected lung produces ultrasound artifacts resulting from the abnormal tissue/gas/tissue interface when US waves penetrate lung tissue. Such artifacts are the basis of lung ultrasound (LUS) application in the clinic (Coley, 2011; Joshi et al., 2019).

Until recently, LUS has been underutilized for evaluation of the lung in pediatrics. Over the years, its applications have changed and its main indications in the pediatric population have expanded. Hence, ultrasound, which was previously used mainly to confirm the presence and nature of pleural effusion as well as to differentiate solid from cystic masses, was later used to evaluate the lung as well. To date, LUS is a frequently used diagnostic tool in daily pediatric clinical practice among clinicians and radiologists, but its application is not yet widely accepted, despite the numerous literature data.

This review analyzed the studies on LUS in pediatrics, published from 2010 to 2020, with the aim of highlighting the usefulness of pediatric LUS. It also described the normal and abnormal appearances of the pediatric lung on ultrasound as well as the benefits, limitations, and possible future challenges of this modality.

## Methods

Our work is a narrative review that included articles from October 2010 to February 2020.

To be included in the review, papers needed to focus on pediatric experience with lung ultrasound to diagnose different pathological conditions.

The bibliographic database named PubMed was chosen to identify potentially relevant documents using keywords “lung children ultrasound” and “lung children echography.” The articles published between October 2010 and February 2020, written in French or English, and concerning the pediatric age including the neonatal age, were taken into consideration.

Papers that did not fit into the conceptual framework of this review or that dealt with the ultrasound experience of adult patients were excluded.

We grouped the studies according to the topic: ultrasound techniques and images; pneumonia; bronchiolitis; pneumothorax; neonatal ultrasound; wheezing.

Furthermore, each section has been divided by year (Supplementary Materials).

## Indications, Limitations, and Use of Lung Ultrasound in the Pediatric Population Over the Last 10 Years

Until recently, LUS has been underutilized for evaluation of the lung in pediatrics. The bony chest and the presence of air within the lungs were thought to interfere with the transmission of US. Currently, it is known that ultrasonography is suitable for the pediatric chest due to the lack of significant subcutaneous fat. Additionally, the pediatric chest wall is only partially ossified, providing additional acoustic windows that are not available in older children or adults. The thymus also allows for adequate acoustic windows for the evaluation of the anterior chest and mediastinum. Non-ossified sternal and costal cartilage which appears relatively hypoechoic in US gradually ossifies with aging thus decreasing acoustic access (Coley, 2011; Joshi et al., 2019).

Over the years, the applications of LUS have changed and its main indications in children have expanded.

### From 2010 to 2015: Lung Ultrasound as a Support Diagnostic Tool

In the first years during which lung ultrasound was used in pediatric clinical practice, the authors were rather skeptical of its use for which they emphasized its limits rather than advantages and limited its use to a few conditions, in clinical practice, mainly in support of other radiological investigations.

The most common indication for LUS was *to evaluate the opacities detected by CXR and the pleural abnormalities* (Coley, 2011; Joshi et al., 2019). For example, it allowed differentiating whether the cause of a completely opaque hemithorax was parenchymal or pleural disease (or both) guiding the appropriate direction of therapy and the eventual thoracic surgery.

Other classic applications of LUS in children – in support of classical radiology – were the *evaluation of mediastinal widening and the study of chest wall lesions*. Specifically, focal masses were studied by LUS to determine their location and whether they were solid or cystic (Coley, 2011; Joshi et al., 2019).

Reviewing the studies performed up to that moment in the pediatric field, several authors (Supakul and Karmazyn, 2013; Tomà and Owens, 2013; Trinavarat and Riccabona, 2014) concluded that the exclusive approach with LUS instead of CXR could only be applicable in case of assessment of large areas of consolidation in contact with the pleural surface or a suitable acoustic window and for the presence of pleural effusions. They (Supakul and Karmazyn, 2013; Tomà and Owens, 2013; Trinavarat and Riccabona, 2014) stated that CXR was the primary imaging modality for evaluating respiratory disease and that chest CT should typically be performed when better pathology characterization or surgical planning was needed.

However, according to other authors (Coley, 2005; Riccabona, 2008; Chira et al., 2011; Mong et al., 2012), LUS could be used in the evaluation of lesions/diseases that appeared to be occult on chest radiography.

### From 2010 to 2015: Lung Ultrasound, More Limits Than Advantages?

Most of the authors (Supakul and Karmazyn, 2013; Tomà and Owens, 2013; Trinavarat and Riccabona, 2014) – reviewing the papers carried out up to 2013–2014 – underlined the limits of the use of LUS in children and the weaknesses of the idea of regularly replacing CXR with LUS.

These limitations included the following: (1) the lack of studies in the pediatric population aimed at evaluating the pathology of the broncho-tracheal airways; (2) the need for contact between the diseased lung portion and the pleural surface and the need to find an adequate acoustic window; (3) the fact that acoustic phenomena are not always directly convertible into images of the human body as direct biomarkers; (4) the difficult sonographic differentiation between consolidation and atelectasis due to the conflicting opinions of the different authors up to that moment (2014). In this regard, Lichtenstein et al. (2009), hypothesized that in infectious lung disease LUS shows an alveolar consolidation with air bronchograms with a specificity of 94% and a sensitivity of 61% for the diagnosis of pneumonia (Lichtenstein et al., 2009; Tomà and Owens, 2013). On the other hand, Riccabona et al. (2008), stated that ultrasound cannot reliably differentiate between atelectasis caused by pneumonia and other causes and that caution is needed about the significance of small areas of subpleural lung consolidation and pleural line abnormalities (Kim et al., 2000; Riccabona et al., 2008; Caiulo et al., 2013). (5) The comparison of LUS was with CXR in the studies performed. Instead, the gold standard for the respiratory disease should be lung CT, which cannot be used habitually for ethical reasons of radiation exposure (Supakul and Karmazyn, 2013; Tomà and Owens, 2013; Trinavarat and Riccabona, 2014); (6) the fundamental dependence on the patient’s clinical information before performing LUS and the usefulness of having the most recent CXR because, according to the authors (Supakul and Karmazyn, 2013; Tomà and Owens, 2013; Trinavarat and Riccabona, 2014), it would allow to guide the study in the area of interest. In addition, other studies (Tomà, 2013a,b; Tomà and Owens, 2013; Trovato et al., 2013a,b; Trovato and Sperandeo, 2013) on a larger population did not confirm the usefulness of using B-lines/vertical artifacts in the differential diagnosis of dyspnea due to poor specificity. These studies did not also find sufficient evidence for the use of LUS as a substitute for CXR in diagnosing pneumonia.

Nevertheless, the same authors of these reviews conducted up to 2014 (Supakul and Karmazyn, 2013; Tomà and Owens, 2013; Trinavarat and Riccabona, 2014), following the ALARA principle, also stated that LUS should be promoted in pediatric respiratory disease as a valuable imaging tool while respecting of its restrictions and limitations that could be overcome by adding diagnostic tool. The author’s further state that it is not important to stick to the old concept of technique choice substitution. Surely the best results are obtained by choosing case by case and by integrating the different tools.

All things considered, such reviews (Supakul and Karmazyn, 2013; Tomà and Owens, 2013; Trinavarat and Riccabona, 2014) have limitations: few articles have been analyzed on pediatric lung disease (Caiulo et al., 2013) and some of them also have a low number of cases. Most of the reported studies were performed on adults in an emergency setting. The objective evaluation of experimental data of studies on the pediatric population and those increasingly produced worldwide has been neglected (Shah et al., 2013).

### Since 2015: Lung Ultrasound and the Reassessment of Its Use

For many years the new LUS applications remained strictly within the confines of adult critical care units. Over the years, several authors (Kim et al., 2000; Copetti et al., 2008; Reissing et al., 2011; Mong et al., 2012; Caiulo et al., 2013) proposed translating this acquired experience in adults for LUS application in children. This trend has prompted a re-evaluation of the classic ultrasound patterns, contemporarily introducing a new sonographic semiotic.

In this regard, over the years, evolving technology and greater understanding of the artifacts of LUS allowed for its greater applicability of the pediatric chest (Kim et al., 2000; Copetti et al., 2008; Reissing et al., 2011; Mong et al., 2012; Caiulo et al., 2013).

The new clinical and preclinical phase studies (Volpicelli et al., 2012; Soldati et al., 2015; Martelius et al., 2016; Sferrazza Papa et al., 2017; Buonsenso et al., 2020) describe the B-lines as vertical hyperechoic reverberations that move in synchrony with the lung and then as key artifacts in the interpretation of the lung ultrasound findings. According to this new point of view, the physiological basis of B lines is represented by a decreased lung aeration and they generally indicate an ultrasound non-specific finding (Volpicelli et al., 2012; Soldati et al., 2015; Sferrazza Papa et al., 2017). However, its quantitative characterization and according to the most recent studies also qualitative can be indicative of an ultrasound pattern more specific.

Multiple B-lines are seen in congestive heart disease, interstitial lung disease, respiratory infections, and neonates. B- lines could also be observed in a limited number of healthy individuals (Volpicelli et al., 2012; Soldati et al., 2015; Sferrazza Papa et al., 2017; Buonsenso et al., 2020).

In this field, Martelius et al. (2016) performed the first study on the pediatric population. They prospectively evaluated 60 patients (0 to 18 years) who underwent chest CT for different clinical reasons and compared the extent of parenchymal changes observed with the number of B-lines on sonography. No pathological findings were detected on CT in 30 cases; in the others, parenchymal changes were seen in the anterolateral regions. The number of B-lines on LUS was found to consistently increase with the growing extent of parenchymal changes on CT. Parenchymal changes on CT associated with a significantly increased B- line count often included ground-glass opacities, interlobular septal thickening, parenchymal bands, and atelectasis. The results of this study suggested that B-lines were considered to be highly non-specific in children and not useful to differentiate pathologic processes of the lung parenchyma. However, the authors proposed LUS as a screening and follow-up tool for estimating the extent of parenchymal changes in children with respiratory symptoms, taking into account that few published data were available on LUS findings in healthy individuals.

Moreover, in a large population with multiple respiratory conditions, one could expect one-third of the lung ultrasound studies to show a B-line pattern in at least one thoracic area, with etiology varying between different age groups. Since this pattern could be attributed to a wide range of conditions, its interpretation cannot withstand analysis of the distribution, extension, and severity of the B-line pattern along with accurate clinical correlation (Sferrazza Papa et al., 2017).

In 2017, Yousef (2017) published a study in which the population was represented by pediatric patients of the cardiac intensive unit undergoing congenital heart surgery. Patients underwent both CXR and LUS in the post-operative time. On LUS, the number of B-lines increased proportionally with the increase in extravascular lung water (EVLW) and progressed to white lungs which are the equivalent of the ground glass appearance on chest X-rays and CT. The results of this study are highly interesting. The authors reported a significant positive correlation between early B-line scores, obtained during the first hours after surgery, and short-term clinical outcomes. The author concluded that in consideration of the inability to see non-superficial lesions, LUS can be used as a complement to the CXR in order to improve patient care (who is at risk to develop or who has already developed cardiogenic pulmonary edema) and reduce accumulated radiation doses.

In the following years, Soldati and other authors (Soldati et al., 2016, 2019; Soldati and Demi, 2017) through studies on physical models clarified the concept of the sonographic interstitial syndrome (SIS). It is a characteristic ultrasound picture of the hyperdense and unconsolidated superficial lung characterized by the presence of multiple vertical, patched, or diffuse artifacts (B lines) that fan out from the lung wall interface (see dedicated paragraph “Imagine Findings”).

In the presence of edema, ARDS, interstitial lung disease, non-consolidating pneumonia, and contusions, part of the lung volume originally occupied by air can be replaced with water, connective tissue, cells, hyaline membrane, or edematous tissue, eventually creating acoustic traps for the US beam containing a medium that is physically (in terms of acoustic impedance) very different from the surrounding environment (air). Vertical artifacts or B-lines are artifactual and not real images and in their variable aspect, indicate a loss of peripheral lung aeration (without tissue consolidation) due to interstitial disease or simply to lung deflation without histological alterations.

The transition from vertical artifacts to consolidation is a continuum with something similar to the transition between the ground glass and consolidation in chest CT, where ground glass is due to thickening of the interstitium and/or the presence of fluid and/or the presence of collapsed areas and/or increased circulation. In a sense, the vertical artifacts have some correspondence with ground glass, even if they are already present in sufficient conditions to create acoustic channels that anatomically do not reach the entity of the ground glass.

Soldati et al. (2016, 2019); Soldati and Demi (2017) through studies on physical models explain how B-lines or vertical artifacts cannot easily differentiate the causes in the absence of an analysis of their appearance. The same authors demonstrated on physical models that the B lines are heterogeneous entities in terms of aggregation and visual structure, the nature of which is linked to the superficial histological characteristics of their wavelength.

Vertical artifacts generated by a fibrotic or inflammatory lung have a different appearance from those generated by cardiogenic edema (see dedicated paragraph “Imagine Findings”).

In the context of the re-evaluation of LUS applications in pediatric clinical practice, different studies, that compared the use of LUS vs. CXR and vs. pulmonary auscultation in the evaluation of pulmonary consolidations and other injuries, had new goals which were as follows: (1) to understand if LUS could be a diagnostic tool not only supporting diagnostic chest X-ray; (2) to reassess the implementation of the LUS as point-of-care ultrasound (PoCUS).

Some authors (Berant et al., 2015; Chen et al., 2015; Zhang, 2015; Coca Pérez et al., 2016; Cox et al., 2017) have re-evaluated the implementation of the LUS as PoCUS intended as bedside ultrasound examination of the patient by the physician in charge mainly in the Pediatric Intensive Units (PIU) and in the Pediatric Emergency Departments (PED).

In this study, LUS was compared to CXR in the diagnosis of pulmonary diseases. The authors evaluated the following: (1) the characterization of non-specific areas of the white lung on chest x-ray; (2) the detection of small subpleural consolidations not detected on chest x-ray; (3) the diagnosis and monitoring of acute pulmonary edema in patients with acute heart disease; (4) the early diagnosis and post-treatment follow-up of respiratory complications in children with acute respiratory disease such as atelectasis and secondary pneumothorax; (5) the guiding to the complex alveolar recruitment maneuver in small patients with acute respiratory distress and areas of atelectasis; (6) the diagnosis and characterization of the pleural effusion when less than 10 ml. Several authors (Berant et al., 2015; Chen et al., 2015; Zhang, 2015; Coca Pérez et al., 2016; Cox et al., 2017) stated that there are limitations to CXR such as poor image quality, presence of artifacts, the time required to obtain the image and exposure to ionizing radiation. It was also shown (Berant et al., 2015; Chen et al., 2015; Zhang, 2015; Coca Pérez et al., 2016; Cox et al., 2017) that CXR is insensitive for detecting small volumes of pleural fluid less than 200 ml and is not able to define the nature of the pleural fluid. Some studies (Chen et al., 2015; Cox et al., 2017) confirmed a sensitivity and a specificity of POCUS for the diagnosis and follow-up of the most common pediatric and neonatal lung diseases higher than that of CXR.

There are also limitations to CT, the gold standard for the diagnosis of respiratory pathology, including its high cost, reduced availability, high radiation exposure, and difficulty in transporting the patient out of the hospitalization unit (Chen et al., 2015; Cox et al., 2017). So, the authors (Chen et al., 2015; Cox et al., 2017) proposed LUS as a convenient, non-invasive, safe, and radiation-free tool that can be quickly performed at the patient’s bed without the need to move, to help in the differentiation of lung diseases and to become a reference instrument for the monitoring of respiratory dynamics and the follow-up of respiratory diseases (Chen et al., 2015; Cox et al., 2017). According to them (Chen et al., 2015; Cox et al., 2017), the clinical information collected at the bedside is essential to guide care quickly and correctly and decrease uncertainty so giving the LUS a fundamental role in the diagnostic and therapeutic process of respiratory diseases.

In a prospective study, Lovrenski et al. (2016) compared LUS with auscultation findings. In children with clinical suspicion of pneumonia, LUS showed positive findings of lung consolidations to a greater extent than auscultation which, on the contrary, was to a greater extent associated with negative findings. According to the authors, moreover, a craniocaudal size of subpleural consolidation of less than 30 mm significantly reduced the possibility of auscultator detection (in approximately 95% of auscultator examinations). In addition, the use of an additional trans-abdominal US approach, along with the standard trans-thoracic approach, could be expected to result in a further increase in US sensitivity for the diagnosis of pneumonia, which was already high (the sign referred to as “dynamic air bronchogram” had a reported positive predictive value of 97% for the diagnosis of pneumonia) (Lovrenski et al., 2016).

Some authors (Copetti, 2016; Lovrenski et al., 2016; Cox et al., 2017; Lovrenski, 2020) based on their review studies, stated that whenever lung US is consistent with clinical and laboratory findings and auscultation, chest radiographs might be avoided. Furthermore, when clinical findings are uncertain, but a classic LUS pneumonic pattern is evident (for example, lung parenchyma consolidation with branched air bronchogram), we should consider avoiding chest radiography, using lung US to monitor the effects of therapy.

In conclusion, studies performed over the last few years report that one of the most valuable aspects of LUS application is its utility in the follow-up of pneumonia helping clinicians to make proper therapeutic adjustments if needed without exposure to radiation. It enables detection of early stages of necrotizing pneumonia by revealing minor areas of necrosis, which often cannot be seen on CXR.

Moreover, it allows a significant reduction in the number of chest CT and CXR in children with necrotizing pneumonia, both early and extensive ones, and sometimes allows even avoidance of chest CT, especially when the clinical course of the disease shows a regression of symptoms and improvement of laboratory findings. Of course, CT should be employed in complicated cases and when the clinical course of the disease is not improving.

Although most studies report that trans-thoracic examination is sufficient, the trans-abdominal (trans-hepatic and trans-splenic) approach can increase the sensitivity of lung US in detecting pneumonia by recognizing patterns other than normal mirror-image phenomenon, which represent a supradiaphragmatic projection of the liver and spleen. (Copetti, 2016; Lovrenski et al., 2016; Cox et al., 2017; Joshi et al., 2019; Lovrenski, 2020).

**Since 2018,** several authors have begun to conduct studies in specific settings - not only in pediatric and neonatal ICUs - with the aim of defining specific ultrasound patterns for each disease with the possibility of creating ultrasound scores for the various pediatric lung diseases. In this way, LUS could be used as a non-invasive clinical marker to evaluate the evolution of a particular acute or chronic lung disease and the response to therapy (see the section “The main fields of application of LUS in pediatrics”).

## Technique and Method of Scanning

As for the scanning technique and method, there was substantial homogeneity among the different studies that were included in this review.

### Lung Ultrasound Scanning Mode

The ribs in neonates and small infants have low mineral content, thus allowing trans osseous scanning, especially in the parasternal region where the ribs are cartilaginous (Shah and Greenberg, 2017; Joshi et al., 2019). This can be done through the trans-sternal and trans-costal approaches.

Although most studies report trans-thoracic examination as sufficient, trans-abdominal (trans-hepatic and trans-splenic) approach can increase the sensitivity of LUS in detecting pneumonia or SIS areas (Copetti, 2016; Lovrenski et al., 2016; Cox et al., 2017; Joshi et al., 2019; Lovrenski, 2020). According to some authors (Joshi et al., 2019), evaluation of the diaphragm, the subdiaphragmatic space as well as the liver and spleen should form part of the protocol as lung consolidation and empyema may be secondary to a liver abscess.

### Probes and Transducers

The type and frequency of transducer used would vary with the age of the patient and the location of the lesion.

Linear transducers with high frequency with a small footprint are preferred to perform sagittal and intercostal scans in neonates and infants. In addition, the linear probe is the best choice for studying the dynamics of breath-dependent motion as well as pleural line abnormalities.

Lower-frequency transducers are used for older children and overweight or obese adolescents (Lovrenski et al., 2016). Curved array transducers are used to insonate between ribs, below the diaphragm, or from the suprasternal notch (Joshi et al., 2019; Strzelczuk-Judka et al., 2019).

In particular, all the studies used a high-resolution linear probe 10 MHz or more. However, the use of both high-frequency linear probes and lower or intermediate frequency linear probes (Zhang, 2015; Lovrenski et al., 2016; Martelius et al., 2016; Song et al., 2017; Strzelczuk-Judka et al., 2019; Tripathi et al., 2019) and low-frequency convex probes have been used in the same studies (Lovrenski et al., 2016; Strzelczuk-Judka et al., 2019; Tripathi et al., 2019).

Two studies (Ho et al., 2015; Yadav et al., 2017) only used the convex probe which, however, did not bring about changes in the execution of the ultrasound investigation and the evaluation of the images.

### Scanning Protocol and Examination Position

Most of the LUS studies in the pediatric population have been performed in critically ill patients with or without respiratory distress in the pediatric emergency departments or pediatric/neonatal intensive care units (Cattarossi, 2014; Zhang, 2015; Song et al., 2017, 2018; Yousef, 2017; Tripathi et al., 2019). Therefore, patients were scanned in the supine position. Only if conditions allowed, they were scanned in sitting or reclining position.

In the case of stable patients, each hemithorax is divided into six regions using two longitudinal lines (anterior and posterior axillary line) and two axial lines (one above the diaphragm and the other 1 cm above the nipples). The lung areas are the anterior (between the sternum and the anterior axillary line), the lateral (between the anterior and posterior axillary lines), and the posterior (between the posterior axillary line and the spine) (Lovrenski et al., 2016; Martelius et al., 2016; Weerdenburg et al., 2016; Yousef, 2017; Strzelczuk-Judka et al., 2019; Lovrenski, 2020).

For a comprehensive examination, the 12 lung areas are sequentially scanned from right to left, cranial to caudal until the diaphragm is revealed, and anterior to posterior.

## Imaging Findings

There has been a complete consensus among all the studies with respect to the imaging criteria required for diagnosing the various respiratory conditions. Most of the studies clarify that US findings are considered physiological and pathological and describe the LUS pathological findings mainly translating the experience gained from the adult population into pediatric clinical practice.

### Normal Pattern of the Lung (Figure 1 and Electronic Supplementary Video 1)

The LUS findings of a normal lung (Copetti et al., 2008; Lichtenstein and Mauriat, 2012; Volpicelli et al., 2012; Lichtenstein, 2014; Saraogi, 2015; Chen et al., 2017; Shah and Greenberg, 2017; Joshi et al., 2019) are represented by:

- **The pleural line** represents the normal lung surface (an intense interface due to high variation of impedance from the chest wall to lung parenchyma) and results in a single smooth regular hyperechogenic line with a thickness of lesser than.5 mm below the rib line, formed by sound waves reflected from the parietal and visceral pleura. A normal pleural line is characterized by the presence of sliding: a “to and fro” movement of lung surface synchronized with respiration (Copetti et al., 2008; Volpicelli et al., 2012; Chen et al., 2015; ShahC et al., 2015; Joshi et al., 2019).

**FIGURE 1:**
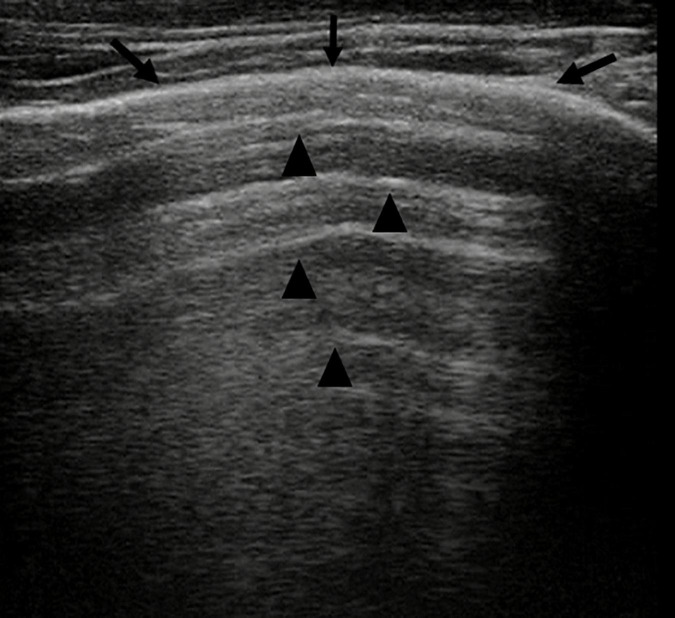
Grayscale lung ultrasound examination (transverse scan between intercostal fields; linear probe with 12 MHz frequency) shows a normal lung ultrasound pattern: hyperechoic, regular, and smooth pleural line with a thickness of less than 0.5 mm (*arrows*), pleural sliding present, and normally represented characterized by “to and fro” movement of lung surface synchronized with respiration (Electronic Supplementary Video 1). Below the pleural line, lung ultrasound imagines show A-lines (*arrowheads*): echogenic horizontal lines parallel and equidistant from each other which indicate the presence of normally aerated lung.

In pathological conditions, the pleural line can be absent or present with a thickness more than.5 mm or with a coarse and irregular appearance with or without evidence of small subpleural consolidation; just as pleural sliding can be absent or poorly represented (Copetti et al., 2008; Volpicelli et al., 2012; Chen et al., 2015; ShahC et al., 2015; Joshi et al., 2019).

- **A-lines,** echogenic horizontal lines parallel and equidistant from each other, which indicate the presence of normally aerated lung. The lung and the soft tissues differ in their acoustic characteristics causing reflection of the ultrasound waves from the lung surface creating reverberation artifacts that are configured in these lines (Copetti et al., 2008; Lichtenstein and Mauriat, 2012; Lichtenstein, 2014; Chen et al., 2015; Joshi et al., 2019).

The search, identification, and evaluation of these US findings of normality are performed in B-mode (Copetti et al., 2008; Lichtenstein and Mauriat, 2012; Volpicelli et al., 2012; Lichtenstein, 2014; Saraogi, 2015; Chen et al., 2017; Shah and Greenberg, 2017; Joshi et al., 2019).

### Pneumothorax (Figure 2 and Electronic Supplementary Video 2)

The accuracy of US as a first-line investigation for detection of pneumothorax (PTX) is almost comparable to the accuracy of CT and far exceeds the accuracy of plain radiographs (Coley, 2011; Volpicelli et al., 2011; Chen et al., 2015; Chen and Zhang, 2015; Zhang, 2015; Coca Pérez et al., 2016; Raimondi et al., 2016; Cox et al., 2017; Joshi et al., 2019).

**FIGURE 2:**
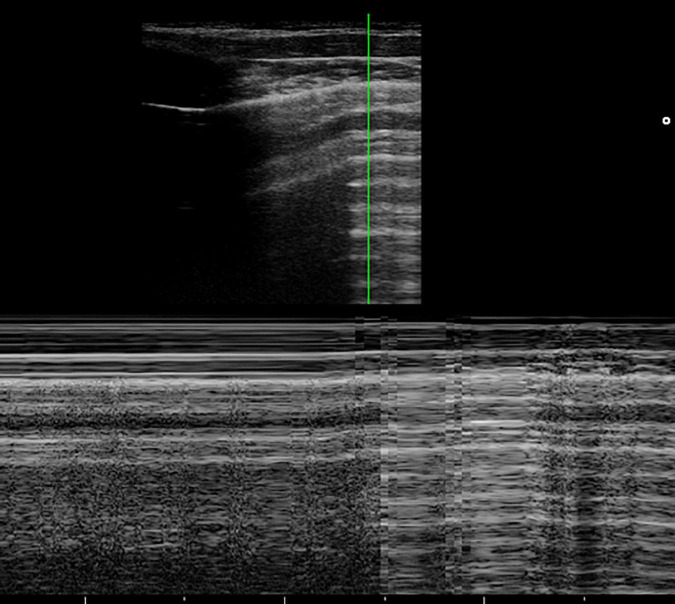
The grayscale lung ultrasound examination (transverse scan between the intercostal fields; linear probe with a frequency of 12 MHz) of a 15-year-old boy with left apical pneumothorax shows the *Barcode sign* identified in M-mode as a transition from the normal lung (*linear granular pattern on the right of the figure*) to the pneumothorax (*linear pattern on the left of the figure*). The grayscale lung ultrasound examination (transverse scan between the intercostal fields; linear probe with a frequency of 12 MHz) of a 16-year-old boy with right apical pneumothorax, shows the *Lung Point sign* identified in B-mode as the point where normal sliding disappears due to the presence of air in the pleural cavity.

According to the US signs of PTX, there is an agreement between the various studies taken into consideration. In particular, it is identified by:

- **Absence of lung sliding** (specificity and sensitivity of 91.1 and 95.3%, respectively) **and absence B-lines**, both identified in B-mode, originating from the visceral pleura (negative predictive value of 99.2–100%).
- **Lung point**, (specificity and sensitivity of 100 and 79%, respectively), identified in B-mode, the point at which normal sliding disappears because of the presence of air in the pleural cavity (Electronic Supplementary Video 2).
- **Double lung point**, (Zhang, 2015), identified in B-mode, whose presence indicates limited pneumothorax so indicating conservative management. Between two lung points, there is no lung sliding or B-line, suggesting separation of visceral and parietal pleura. Laterally to the points, the pleural sliding and B-line signs are evident. Both lung points move simultaneously with respiration.
- **The barcode sign**, identified and studied in M-mode, occurs as there is no motion of the chest wall and no motion of the lung due to the presence of air in the pleural cavity. This is seen as multiple parallel horizontal lines resembling a bar code. It is represented by the transition from the normal lung (linear granular pattern) to the pneumothorax (linear pattern) (Figure 2).

### Pleural Effusion (Figures 3, 4A)

The role of LUS in confirming the presence of pleural effusion is well established (Coley, 2011; Prithviraj and Suresh, 2014; Berant et al., 2015; Cox et al., 2017; Joshi et al., 2019; Toma et al., 2019).

**FIGURE 3:**
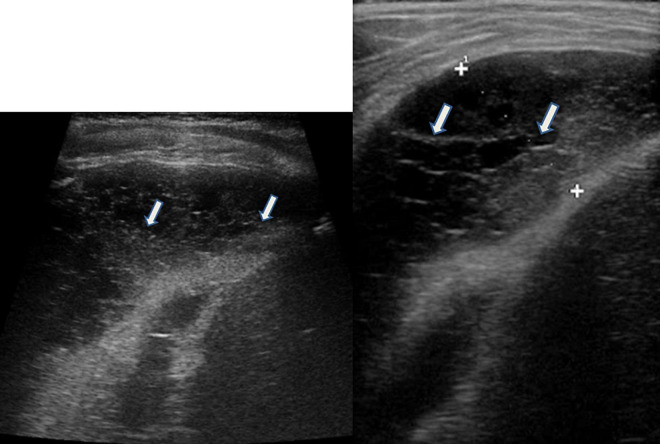
Grayscale lung ultrasound examination (transverse scan between intercostal fields; linear probe with 12 MHz frequency) of a 5-year-old child with pneumococcal pleuropneumonia (complicated by pleural empyema), shows exudative pleural effusion, with internal echoes non-homogeneously distributed: it is fibrinous, plural-septate, and concamerated and with thickened septa (*arrows*).

**FIGURE 4:**
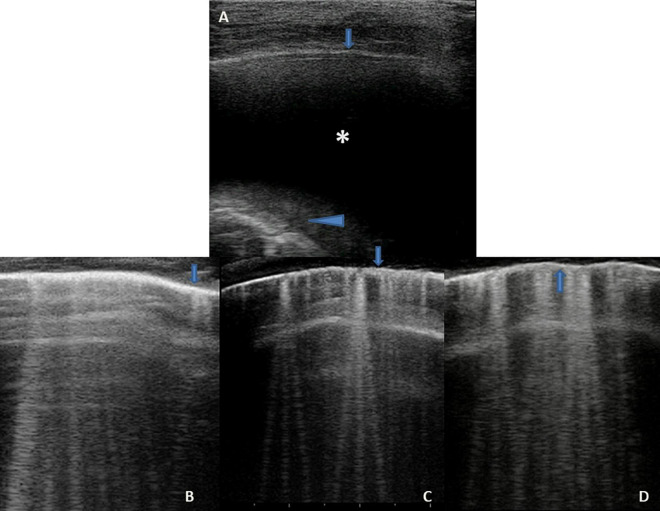
Grayscale lung ultrasound examination (transverse scan between intercostal fields; linear probe with 12 MHz frequency) of a 10-year-old child with congestive heart disease resulting from untreated rheumatic carditis, shows in Panel **(A)**, on the basal posterior-lateral fields on the right, an anechoic pleural effusion (*asterisk*) that appears like an anechoic space between the parietal (*arrow*) and visceral pleura (*arrowhead*) below which lung appears atelectasis. Panels **(B,C)** show the SIS pattern of the upper right and left fields, respectively; Panel **(D)** shows that of the right lower/basal fields: the pleural line is regular (*arrows*); vertical artifacts or B-lines are separated, laser-like artifacts, with gravitational course spreading from the pleural line to the bottom of the screen and they show an internal sequence of alternating horizontal bands and homogeneous distribution in the affected spaces. Ultrasound picture compatible with the septal pattern of early CPE (Soldati and Demi, 2017; Soldati et al., 2019). CPE, cardiogenic pulmonary edema; SIS, sonographic interstitial syndrome.

Lung ultrasound (LUS) is a useful and safe tool for evaluating pleural effusion because it allows the distinction between effusion and lung consolidations and has greater accuracy in detecting pleural effusion than bedside CXR. CXR can detect the presence of pleural effusion in patients in an orthostatic position only if the volume of the effusion is at least 200 mL and the sensitivity of this method decreases in the supine position, while LUS can detect effusions as small as 10-20 mL (Coley, 2011; Prithviraj and Suresh, 2014; Berant et al., 2015; Cox et al., 2017; Joshi et al., 2019; Toma et al., 2019).

Lung ultrasound (LUS) evaluation of a patient in a sitting or standing position is better because it allows for more precise quantification of pleural effusion and the visualization of small amounts of fluid in the pleural cavity. In this position, the free fluid will collect in decline space while it will be found in a posterior location with the patient supine.

In addition, ultrasound allows the identification of adjacent structures: chest wall, hemidiaphragm (over the liver or spleen), and visceral pleural surface. This is important, especially in the case of an invasive procedure, in order to avoid organ injury (Coley, 2011; Prithviraj and Suresh, 2014; Berant et al., 2015; Cox et al., 2017; Joshi et al., 2019; Toma et al., 2019).

Unlike CXR, US can provide information on the nature of the pleural fluid. It is superior to CT in characterizing the nature of pleural fluid collections and can help guide percutaneous drainage. However, in complicated and refractory cases CT may be a better option, especially if surgery is planned (Coley, 2011; Prithviraj and Suresh, 2014; Berant et al., 2015; Cox et al., 2017; Joshi et al., 2019; Toma et al., 2019).

Pleural effusions have a different echogenicity on US depending on the underlying causes (Coley, 2011; Prithviraj and Suresh, 2014; Berant et al., 2015; Cox et al., 2017; Joshi et al., 2019; Toma et al., 2019).

- **Transudative effusions** (Figure 4A) appear as a space (usually anechoic) between the parietal and visceral pleura that changes depending on the patient’s position. The lung appears with varying degrees of compression and, depending on the amount of fluid, also moves with breathing (“sinusoid sign”) or heartbeat (“pulse sign”).
- **Exudative effusions** (Figure 3) show echoes that suggest the presence of debris (cell, blood fibrin). Fibrin can be observed in exudative effusions but the amount, distribution, and organization in septa or loculi differ from patient to patient, depending on the cause of the effusion and the time from the onset.

The additional presence of a thickened pleura or pulmonary consolidation with dynamic air bronchogram may suggest **the infectious nature of the pleural effusion** (Coley, 2011; Prithviraj and Suresh, 2014; Berant et al., 2015; Cox et al., 2017; Joshi et al., 2019; Toma et al., 2019).

The presence of a diffuse sign of lung congestion (B-lines or vertical artifacts) suggests **transudative effusion during heart failure** (Yousef, 2017) (Figure 4).

Although various US methods for quantifying the volume of pleural effusions have been described, all require several measurements. Many authors believe that knowing the exact amount of fluid is of limited usefulness in clinical practice.

A qualitative approach may be useful, summarized in the Table 1 below (Prina et al., 2014). Additionally, LUS can help estimate the effect of pleural effusion on lung parenchyma by allowing visualization of different degrees of collapse (Prina et al., 2014).

**TABLE 1:** Lung ultrasound assessment of pleural fluid and its estimate of volume.

Quantification	Ultrasound visualization	Volume estimation mL
Minimal	Costophrenic angle	≤ 100
Small	Range, one probe	100–500
Moderate	Range, two probes	500–1.500
Large or Massime	Range, three or more probes	> 1500

### Lung Consolidations (Figures 5–8)

**FIGURE 5:**
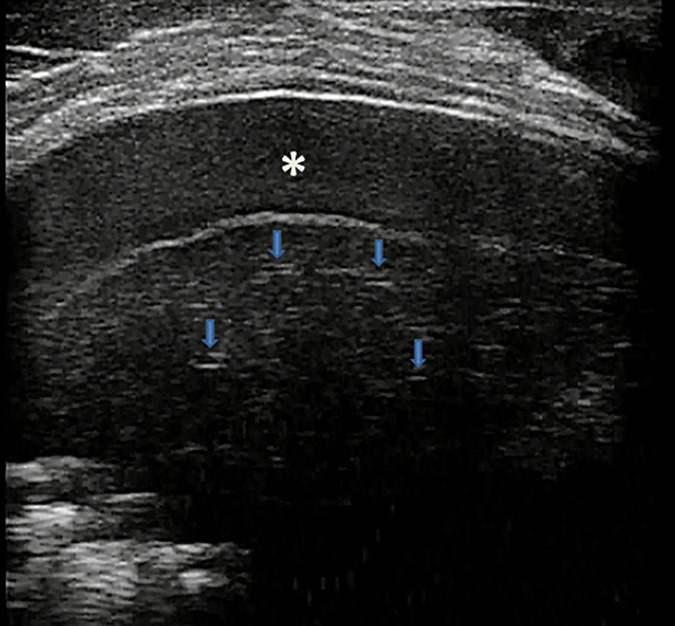
Grayscale lung ultrasound examination (transverse scan between intercostal fields; linear probe with 12 MHz frequency) of a 7-year-old boy with bacterial lobar pneumonia, shows hepatized subpleural consolidation with fluid bronchograms (*arrows*) and fibrinous inflammatory reactive pleural effusion (*asterisk*).

**FIGURE 6:**
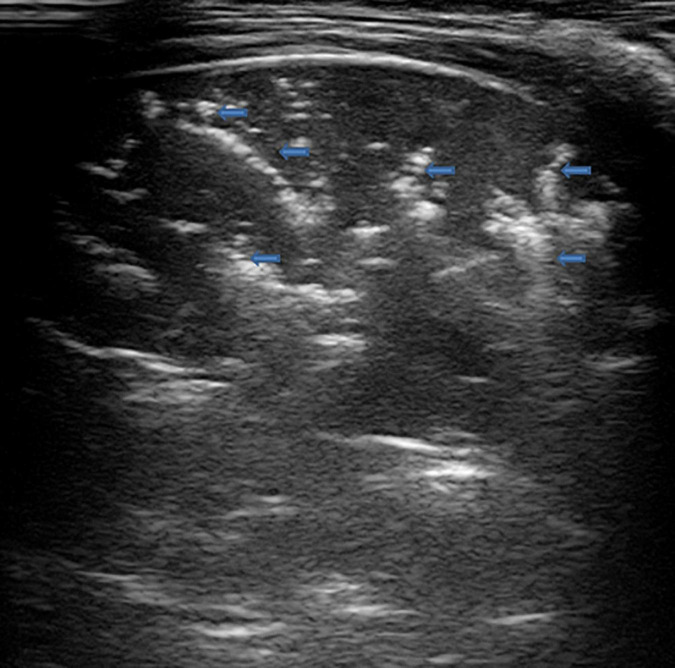
Grayscale lung ultrasound examination (transverse scan between intercostal fields; linear probe with 12 MHz frequency) of a 5-year-old boy with bacterial pneumonia lobar, shows subpleural consolidation of an inflammatory/infectious nature with numerous elements of surface dynamic arborized bronchograms (*arrows*) – (Electronic Supplementary Video 3) and deep fluid bronchogram.

**FIGURE 7:**
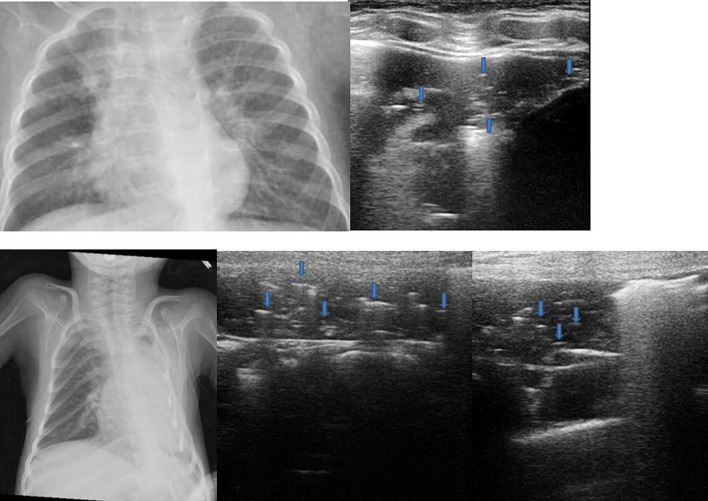
The upper part of the figure shows chest X-ray and lung ultrasound images of a 7-year-old girl with uncontrolled asthma and an ongoing severe asthma attack, with no clinical or laboratory signs of infections. The chest X-ray shows non-specific areas of reduced transparency in the right apical and right basal sites. Grayscale lung ultrasound examination (transverse scan between intercostal fields; linear probe with 12 MHz frequency) shows – on the right paracardiac area – subpleural consolidation with hyperechoic images of air bubbles (static aerial bronchograms, *arrows*) that do not move inward during breathing and are parallel to each other. Their presence is associated with the identification of atelectasis, supported by the clinical and laboratory context of the patient being examined. The lower part of the figure shows chest X-ray and lung ultrasound images of a 4-year-old girl with neuromuscular pathology in nocturnal non-invasive ventilation with ongoing respiratory exacerbation without laboratory and clinical signs of infections. The chest X-ray shows a completely white left lung not well definable. Grayscale lung ultrasound examination (transverse scan between intercostal fields; linear probe with 12 MHz frequency) shows the entire left lung’s subpleural consolidations with hyperechoic images of air bubbles (static aerial bronchograms, *arrows*) static and parallel to each other. Their presence is associated with the identification of left pulmonary atelectasis. The ultrasound suspicion is supported by the clinical and laboratory context of the patient being examined.

**FIGURE 8:**
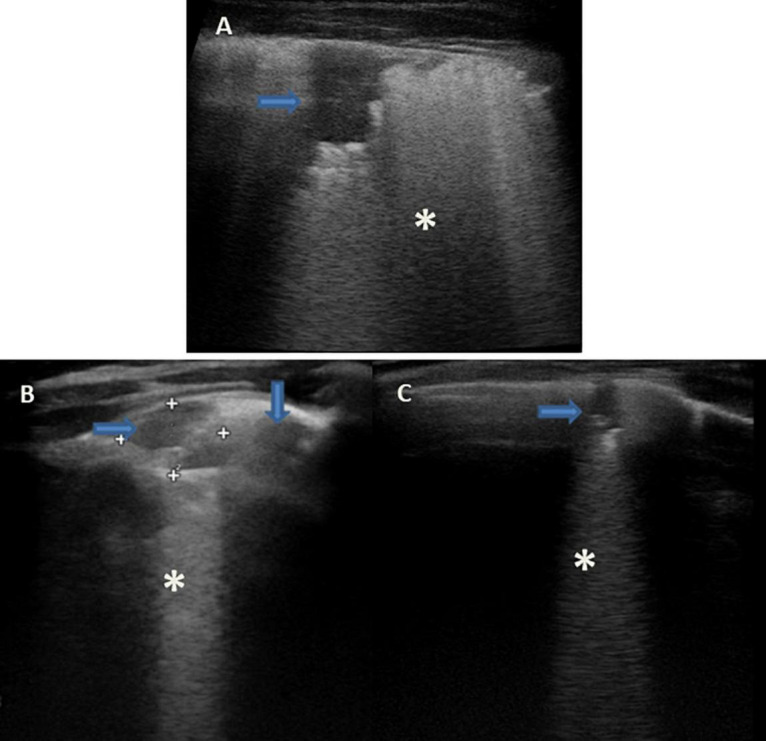
Grayscale lung ultrasound examination (transverse scan between intercostal fields; linear probe with 12 MHz frequency) of children with viral lower respiratory tract infection [**(A)**: 2-year-old boy with H1N1 Influenza pneumonia; **(B,C)**: 8-month-old infant with Respiratory Syncytial Virus bronchiolitis]. Lung ultrasound findings show sonographic interstitial syndrome (SIS) with areas of the white lung with multiple, coalescent vertical artifacts (B-lines, asterisks) and small subpleural consolidations (hypoechoic areas, *arrows*) less than 1 centimeter in size associated with areas of “white lung” or confluent B-lines (*asterisks*).

When a lung area loses its normal aeration due to an inflammatory event or collapse of the airways, a consolidation is created in the lung parenchyma, and the displayed image is a real image and not an artifact (Coley, 2011; Chen et al., 2015; Perez et al., 2015; Lovrenski et al., 2016; Claes et al., 2017; Cox et al., 2017; Joshi et al., 2019; Lovrenski, 2020). In fact, within the consolidated area we can recognize:

- **Hepatization** (Figure 5), which is defined as that area of the lung without air that mimics the appearance of the liver. In consolidations, alveoli are replaced with fluid and inflammatory debris resulting in hepatization of the lung that is characterized in the US by a hypoechoic aspect and Doppler vessels (Chen et al., 2015; Cox et al., 2017; Joshi, 2019).
- **Dynamic air bronchograms** (Figure 6; Electronic Supplementary Video 3), the branching echogenic foci within a consolidation, represent the residual air within the bronchi and some of the alveoli. They are represented by hyperechoic images of air bubbles moving within the airways with a centrifugal respiratory progression in inspiration. The presence of dynamic air bronchogram is associated with pneumonia identification in approximately 70–97% of cases and with the exclusion of an atelectasic area and/or bronchial obstruction (Lichtenstein et al., 2009; Chen et al., 2015; Cox et al., 2017; Joshi, 2019; Lovrenski, 2020).
- **Fluid or mucus bronchograms** (Figure 5), identified by ultrasound in B-Mode and by Color-Doppler, are represented by echo-free tubular structures without any perfusion signal. The air in the bronchi is replaced by fluid. Their presence is associated with the identification of pneumonia (Chen et al., 2015; Joshi, 2019; Lovrenski, 2020).
- **Normal branching pattern of vessels within the consolidated lung**, identified by ultrasound in B-Mode and by Color-Doppler, is useful for differentiating a lung consolidation air from a mass (Chen et al., 2015; Joshi, 2019).
- **Linear, parallel, and static bronchograms** (Figure 7) are hyperechoic images of air bubbles that do not move inward during breathing and are parallel to each other. Their presence is usually associated with the identification of atelectasis. In fact, in atelectasis, the lung appears hypoechoic, triangular in shape with crowding of the bronchi due to loss of lung volume. Only a few of these can have air within them (Chen et al., 2015; Perez et al., 2015; Joshi, 2019; Lovrenski, 2020).
- **Extremely small subpleural consolidations** (Figure 8) with sizes of less than 5–0.1 mm with or without adjacent single or confluent B-lines, whose presence is associated with abnormal ventilated areas or Viral pneumonia or Bronchiolitis (Lovrenski et al., 2016; Lovrenski, 2020).
- **Areas of breakdown/small areas of lung necrosis and lung abscess.** The small areas of lung necrosis appear as areas of decreased echogenicity with no color Doppler flow within a region of pulmonary consolidation. Larger abscesses can develop a thick wall and air-fluid levels may be seen if there is cavitation or if the abscess communicates with the bronchial tree. They are commonly seen in staphylococcal cases of pneumonia, where pneumatoceles can also occur, as well as in acute necrotizing cases of pneumonia (Coley, 2011; Claes et al., 2017; Joshi, 2019).

### Sonographic Intesitial Syndrome (Figures 4, 9)

Coley, 2011; Cattarossi, 2014; Chen et al., 2015; Martelius et al., 2015; Perez et al., 2015; Sferrazza Papa et al., 2017; Yousef, 2017; Joshi, 2019; Soldati et al., 2019; Buonsenso et al., 2020.

**FIGURE 9:**
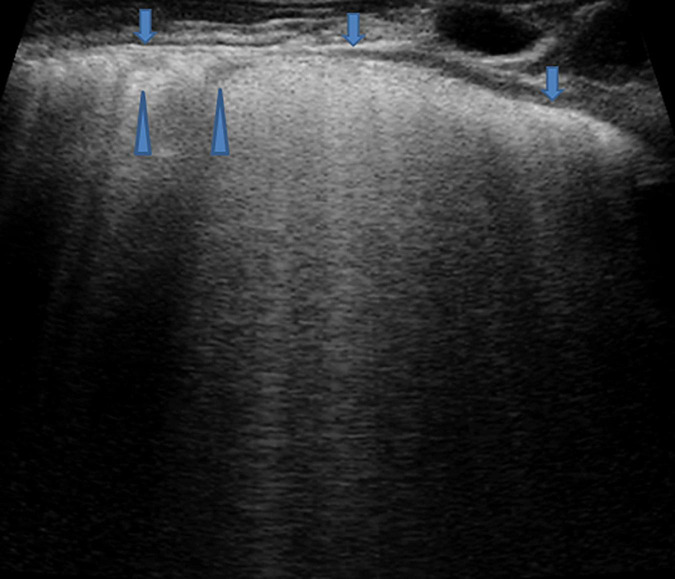
Grayscale lung ultrasound examination (transverse scan between intercostal fields; linear probe with 12 MHz frequency) of a 4-year-old boy with viral pneumonia – due to Coronavirus (non-COVID-19), Bocavirus, and Metapneumovirus coinfection- requiring respiratory assistance with High- flow nasal oxygen at the pediatric department. It shows sonographic interstitial syndrome (SIS) which is characterized by blurred, uneven, coalescent B-lines and white lung; irregular pleural line *(arrows);* reduced pleural sliding; multifocal inhomogeneous involvement; subpleural microconsolidations (generating pseudo-B-lines) *(arrowheads).*

Vertical artifacts of B-lines are seen as vertically oriented artifacts and indicate an abnormality in the interstitial or alveolar compartment. They extend from the pleural line to the edge of the screen.

B-lines or vertical artifacts, in their variable appearances, indicate a loss of peripheral lung aeration (without tissue consolidation, therefore indicating an artifactual image) due to interstitial disease or simply to lung deflation without histologic changes. However, B-lines could not be sufficient to differentiate the causes without an analysis of their appearance.

The fetal lung has a high fluid content hence B-lines can be seen even on the first day of life in neonates without respiratory distress and they usually disappear by the third day. Furthermore, B-lines can also be seen in healthy individuals especially in newborns and/or infants (Buonsenso et al., 2020). B lines are also seen to a greater extent in pulmonary edema, interstitial lung disease, infections, lung contusion, and atelectasis.

Recent studies (Soldati et al., 2019; Buonsenso et al., 2020) have shown that the vertical artifacts have a different appearance depending on the underlying cause: for example, those generated from a fibrotic lung have a different appearance from those generated by cardiogenic edema. Several authors (Soldati et al., 2019) are currently trying to understand and interpret the causes of these differences by performing preclinical studies (e.g., vertical artifacts can be thicker, brighter, and longer than others, each bearing different acoustic information).

In recent years, Soldati et al. (2019) generated a different point of view, demonstrating on physical models that B-lines are heterogeneous entities in terms of aggregation and visual structure, the nature of which is linked to the superficial histologic characteristics of the lung.

Therefore, rather than simply developing algorithms to count artifacts, it could be much more useful to develop new software that, with the help of artificial intelligence, is able to extract quantitative and qualitative information from the vertical artifacts.

- **Cardiogenic origin** (Figure 4) can be characterized by: typical B-lines or vertical artifacts (laser-like, bright) with the septal disposition (early stage); modulated B-lines (early stage); regular pleural line; the presence of pleural sliding; diffuse pulmonary involvement without spared areas (bilaterally); absence of consolidations, pleural nodules or pleural irregularities. Furthermore, they are all characteristics that can take on a dynamic aspect depending on the patient’s position, the effects of the exercise and therapy (Martelius et al., 2015; Perez et al., 2015; Sferrazza Papa et al., 2017; Yousef, 2017; Joshi, 2019; Soldati et al., 2019; Buonsenso et al., 2020).
- **Pneumogenic origin** (Figure 9) can be characterized by: unusual septal disposition of B-lines; blurred, uneven, coalescent B-lines and white lung; non-modulated B-lines or pseudo-B-lines; irregular pleural line; reduced pleural sliding; monofocal or multifocal, patchy or inhomogeneous involvement; consolidations, subpleural nodules or micronodules (generating pseudo-B-lines). Furthermore, the US features do not have dynamic aspects and do not change with the patient’s position, therapy, or movements (Soldati et al., 2019; Buonsenso et al., 2020).

In the context of neonatal pathology, however, the differential diagnosis of the interstitial syndrome commonly falls between the respiratory distress syndrome (RDS) and Transient Tachypnea of Newborn (Joshi, 2019) (see specific section on the neonatal disease).

## The Main Fields of Application of Lung Ultrasound in Pediatrics

### Pneumothorax

The detection of PTX is a growing interest section within pediatric PoCUS even if most of what is reported is based on descriptive cases of retrospective works. Pneumothorax is defined as the pathological presence of air between the parietal and visceral pleura resulting in collapsing of the lung. In its extreme degree, tension PTX is a life-threatening event leading to increased intrathoracic pressure, elevated central venous pressure, and decreased venous return with consequent reduced cardiac output, bradycardia, and ultimately cardiac arrest. Symptoms of PTX may be extremely variable including chest pain, shortness of breath, cough, and increases in heart rate or breathing until respiratory failure. PTX is traditionally classified as spontaneous, traumatic, or iatrogenic and its diagnosis is based on a combination of clinical suspicion along with supporting imaging studies. Computed tomography remains the gold-standard imaging test in the evaluation of PTX, but it is limited by its high radiation exposure, especially in pediatric age, and the need of transporting critically ill patients. So, the most widely used method for bedside evaluation is CXR even if its diagnostic sensitivity is well known to be limited in the supine position with a small pneumothorax (Volpicelli, 2011). These are the reasons why POCUS is getting an ongoing interest in the diagnosis of PTX, although its application in pediatric age is once again deriving mainly from observations conducted on the adult population (Volpicelli, 2011; Volpicelli et al., 2012; Chen and Zhang, 2015; Liu et al., 2017).

Lung ultrasound (LUS) diagnostic accuracy for PTX has been confirmed and can even reach 100% in sensitivity, specificity, positive and negative predictive value (Volpicelli et al., 2012; Raimondi et al., 2016). The diagnosis of PTX by LUS is as reliable as conventional CXR in neonatal age and even more sensitive especially for small PTXs (Deng et al., 2020). Lung ultrasound can help with rapid and timely diagnosis and thus in bedside treatment (Deng et al., 2020) and PTX can even be earlier detected by applying LUS rather than by using only X-ray (Szymońska et al., 2019); furthermore, LUS application in the follow-up of diagnosed PTXs significantly reduces the CTXs performed (Szymońska et al., 2019) and limits the exposure to ionizing radiation (Szymońska et al., 2019; Deng et al., 2020).

There may be some limitation in the ability of LUS to assess the volume of PTX that would be required for choosing surgical vs. conservative treatment. Although there is not a close correlation between the extension on the chest wall and the volume of the intrapleural air, the localization of the lung point in the supine patient allows predicting the extension of PTX through a semi quantification of the volume: large and small PTX (Volpicelli, 2011).

Lung ultrasound (LUS) can therefore be definitely considered the novel approach for PTX evaluation, due to the advantages of timeliness and its high accuracy and reliability.

For the ultrasound diagnostic criteria of pneumothorax (Volpicelli, 2011; Volpicelli et al., 2012; Chen and Zhang, 2015; Liu et al., 2017) see the specific section in “Imaging findings.”

### Neonatal Respiratory Diseases

In the past years, neonatal respiratory diseases have been a diagnostic dilemma for the clinician due to the low sensitivity and specificity of their signs and symptoms and the CXR has not always been able to solve the diagnostic challenge since inter- and intra-observer variability has always been wide.

Lung ultrasound (LUS) may be useful in detecting congenital lung diseases such as pulmonary sequestration, congenital pulmonary airway malformation, and congenital diaphragmatic hernia. Although most of these congenital conditions are detected *in utero* and CT is needed for surgical road-mapping, it is not uncommon to find them accidentally during an abdominal US exam or when referral diagnosis points to another pulmonary condition, such as pneumonia (Lovrenski, 2018; Yousef et al., 2018).

In recent years, LUS is becoming a useful tool in neonatal intensive care units (Avery et al., 1966; Persson and Hanson, 1998; Lichtenstein et al., 2004; Bober and Swietliński, 2006; Copetti and Cattarossi, 2007; Copetti et al., 2008; El-Malah et al., 2015; Liu et al., 2015a; Kurepa et al., 2018; Raimondi et al., 2018; Corsini et al., 2019; Deng et al., 2020) with a variety of differential diagnoses including RDS, transient tachypnea of the newborn, meconium aspiration syndrome, neonatal pneumonia, pulmonary hemorrhage, pneumothorax and bronchopulmonary dysplasia (Lovrenski, 2020).

Some authors (Cattarossi et al., 2010; Cattarossi, 2014; Lovrenski, 2020) have supported the validity of LUS to follow the dynamics of interstitial lung fluid clearance in the postnatal hours, to accurately detect the presence of interstitial and/or alveolar fluid in RDS with ultrasound images that are in full concordance with the clinical course of RDS, but not with x-ray. Lung US showed a sensitivity of 95.6% and a specificity of 94.4%, with a positive predictive value of 91.6% and a negative predictive value of 97.1% for RDS, and a sensitivity of 93.3% and a specificity of 96.5% with a positive predictive value of 96.5% and a negative predictive value of 93.4% for transient tachypnea of the newborn in a more recent article (Liang et al., 2018).

Some authors (Cattarossi et al., 2010; Cattarossi, 2014; Liang et al., 2018) strongly suggest the use of LUS in neonatal age as a first-line imaging technique. The main potential of LUS in the neonatal intensive care unit lies in a dynamic follow-up of changes in the pulmonary condition of neonates; in these cases, LUS can help neonatologists in decision-making in this more vulnerable group of children. Nevertheless, the combination of diagnostic modalities in neonatal intensive care units is essential (Lovrenski, 2020). Chest X-rays are still needed to detect the exact position of lines and tubes, as well as air-leak syndromes (especially pneumomediastinum, pneumopericardium, and interstitial emphysema).

The most encountered applications of LUS in the context of neonatal pathology are RDS and transient tachypnea of the newborn (TTN):

Respiratory distress syndrome (RDS) is one of the most frequent pathological conditions of a newborn. It can occur at birth or in the first hours of life with tachypnea (respiratory rate over 70), dyspnea, gasping, hypotension, pallor, and tachycardia and it moves toward a usual resolution between the second and the fourth day of life. Its principal risk factors are prematurity, because of the lack of surfactant (which begins to be produced from the 24^th^ gestational week), and gestational diabetes, because of fetus hyperinsulinism that interferes with glucocorticoids axes and with surfactant production. The condition is traditionally diagnosed by CXR that shows typical ground glass images.

By LUS, B lines associated with irregularity of the pleural line and subpleural consolidations can lead to the “white lung” condition frequently observed in neonates affected with RDS. A debate about the real concordance between CXR and LUS is ongoing in the literature (Bober and Swietliński, 2006; Copetti et al., 2008; El-Malah et al., 2015; Liu et al., 2015b; Blank et al., 2018; Corsini et al., 2019), although Corsini et al. (2019) described a concordance between LUS and X-Ray of 91%. They also made a comparison in terms of diagnosis time: almost 9 min for LUS vs. 50 min for CXR. Moreover, they proposed a theoretical training of 2 h for novice sonographers and a 30 min hands-on training. The sonographers performed LUS with a successive blinded evaluation by an expert sonographer and a perfect concordance between CXR and LUS was described after 25 performed exams.

Transient tachypnea of newborns (TTN) is a postnatal condition due to a delay in fetal fluid clearance. Principal risk factors are gestational diabetes and cesarean section, considering that in vaginal delivery there is lung compression which would remove excess fluid (Avery et al., 1966; Persson and Hanson, 1998). Kurepa et al. (2018) described the presence of B-lines (not white lung), thickened pleural line, and presence of a typical sign: the double lung points which consists of a straight difference in upper (no B lines) and lower (presence of B lines), in neonates with TTN (Kurepa et al., 2018).

Differential diagnosis between RDS and TTN is not always easy on CXR, but it can allow physicians to differ clinical strategies: the administration of surfactant is performed in RDS while in TTN only ventilation is sufficient. Double lung point has a high sensitivity (although it is reported to vary from 76.6 to 100%) and specificity (100%) in TTN (Lichtenstein et al., 2004; Copetti and Cattarossi, 2007).

**Differentiating RDS from TTN**

**Table:** 

	RDS	TTN
B Lines	Bilateral confluent B-lines No double lung point	Very compact B lines in the inferior pulmonary fields, not so compact in superior lung field - “Double Lung point”
Pleural Line	Thickened and irregular	Normal regular echogenic
Evolution of B Line	Persists, no change even after surfactant	Disappears by day two coinciding with clinical improvement
Lung Consolidation	Associated lung consolidation may be seen	No lung consolidation

RDS: Respiratory Distress Syndrome; TTN: Transient Tachypnea of Newborn (Joshi, 2019).

- *Pneumothorax (PTX)* can also be detected in newborns. Deng et al. (2020) described a cohort of 86 newborns diagnosed PTX by LUS and CXR. In neonates, LUS was more sensitive and more specific for early detection of PTX. Indeed, the absence of B lines, absence of sliding sign, and presence of lung point reached a sensitivity of 100, 100, and 94%, respectively (Deng et al., 2020).
- *Atelectasis* can also be studied by LUS; it is usually described in newborns under mechanical ventilation with no sufficient pressure (Kurepa et al., 2018).
- *Meconium aspiration syndrome* usually shows an LUS finding characterized by one or more consolidations with associated thickened pleura and air bronchograms non-uniformly distributed (Kurepa et al., 2018).

### Pneumonia

Pneumonia remains the leading cause of death globally in children under the age of five.

The first study about the role of LUS in the diagnosis and management of pneumonia was conducted by Caiulo et al. (2013). It’s a single-blind observational study performed on 102 children evaluated in a PED, with the aim of evaluating LUS findings both in the diagnosis and in the follow-up of pneumonia and comparing the sensitivity and specificity of LUS vs. CXR. The authors proved that LUS is a simple and reliable imaging tool, not inferior to CXR in identifying pleuropulmonary lesions in children with suspected pneumonia.

However, according to Tomà (2013a), the use of LUS as a diagnostic tool for infectious respiratory diseases in children would not have been safe if based on the criteria used in studies that involved adult patients. Don et al. (2013) also suggested that to understand the role of LUS in the diagnosis of pediatric pneumonia, further studies with larger, multicenter test samples would be needed, since, until 2014, only a few studies with small sample sizes have been published (Gargani et al., 2012; Shah et al., 2013).

The study of Reissig and Copetti (2014) reviewed the various studies performed up to then on the pediatric population and defined the US characteristics of pneumonia as a hypoechogenic area with: poorly defined borders, presence of B-lines at the far-field margin, less echogenic pleural line in the area affected by the lung consolidation and reduced or absent lung sliding. Furthermore, branching echogenic structures representing air bronchograms were described in the area of the infected zone in the context of consolidations.

Subsequently, several studies that focused on the pediatric population were published in the literature (Copetti and Cattarossi, 2008; Reissig et al., 2012; Chiappini et al., 2013; Audette and Parent, 2016; Milliner and Tsung, 2017; Pervaiz et al., 2018; Xin et al., 2018). The most used study model was the prospective one, but we also took into consideration the other studies whose scientific validity is lower (case report, letter to editor, commentary, meta-analysis, and reviews). In the present review, 27 studies (Gereige and Laufer, 2013; Shah et al., 2013; Esposito et al., 2014; Liu et al., 2014; Reali et al., 2014; Chavez et al., 2015; Iorio et al., 2015; Pereda et al., 2015; Urbankowska et al., 2015; Ambroggio et al., 2016; Claes et al., 2016, 2017; Guerra et al., 2016; Hajalioghli et al., 2016; Ianniello et al., 2016; Jones et al., 2016; Ključevšek, 2016; Boursiani et al., 2017; Brendan et al., 2017; Ellington et al., 2017; Man et al., 2017; Yadav et al., 2017; Yilmaz et al., 2017; Biagi et al., 2018; Chen Z. et al., 2018; Farhan et al., 2018; Xiao et al., 2018; Zhan et al., 2018; Lissaman et al., 2019; Najgrodzka et al., 2019; Tsou et al., 2019; Yan et al., 2020) evaluated LUS in terms of sensitivity and specificity regarding the diagnosis of pneumonia. The individual studies’ sensitivity ranged from 87 to 100 (94%; IQR: 89–97%) and specificity ranged from 85 to 100% (with an average of 94%; IQR: 86–98%).

Among the 27 studies, 8 of these (Shah et al., 2013; Liu et al., 2014; Reali et al., 2014; Boursiani et al., 2017; Balk et al., 2018; Biagi et al., 2018; de Souza et al., 2019; Lissaman et al., 2019) compared sensitivity and specificity of LUS vs. CRX. CXR sensitivity ranged from 82 to 95, while CXR specificity ranged from 90 to 100. These studies suggest that LUS examination can detect lung consolidation and the other ultrasound features of pneumonia in children with the similar accuracy and reliability as chest radiographs with the benefits of no exposure to ionizing radiation and savings in cost and time, both in diagnosis and in follow-up.

Furthermore, several studies have demonstrated the superiority of LUS in identifying even minimal pleural effusion that occurs with pneumonia (Shah et al., 2013; Reali et al., 2014; Balk et al., 2018; de Souza et al., 2019) with a sensitivity of LUS that is higher compared to CXR.

One variable that influenced the specificity is the size of the lesion. In fact, in the work of Shah et al. (2013) the specificity rises from 89 to 97% in children with consolidation greater than 1 cm. According to Biagi et al. (2018), the specificity reaches 98.4% when there are >1 cm consolidations. Another variable regarding sensitivity and specificity seems to be age. According to Liu et al. (2014), a large area of lung consolidation with irregular margins had 100% sensitivity and 100% specificity for the diagnosis of neonatal pneumonia.

Two studies deviate from the results of the previous ones. In one study, Lissaman et al. (2019) reported that LUS sensitivity was 91% (95% CI: 78 to 98%) and specificity was 68% (95% CI: 54 to 80%). However, in this regard, the sonographers were a first-year pediatric emergency medicine fellow and a final-year medical student, both without prior US experience but trained specifically for this study by an emergency physician with 5 years of POCUS experience and a Diploma in Diagnostic Ultrasound. In the other study (Biagi et al., 2019) the diagnosis of consolidations by LUS showed a high sensitivity of 93% but low specificity (14% for expert operators and 25% for novice operators). However, in this study, although CXR was used as the gold standard, LUS and CXR were not always performed consecutively and changes in the lung disease process may have occurred due to the time elapsed between the two imaging studies. Also, the sample size is very small, in which only 23 patients were recruited.

Although lung CT is considered the gold standard for the diagnosis of pneumonia, for reasonable ethical reasons no studies have subjected patients to lung CT except in case of clinical need. In most studies, LUS was therefore compared with CXR, the widely used diagnostic tool to diagnose pediatric Community-Acquired Pneumonia (pCAP).

On the other hand, the other authors, evaluated the diagnostic accuracy of *LUS in the diagnosis of pneumonia by comparing it with clinical diagnosis defined by WHO guidelines* (Chavez et al., 2015). They showed that the WHO algorithm did not agree with the results of POCUS in over a third of children and had an overall low performance compared to point-of-care ultrasound to identify lung consolidation. One of the future challenges of LUS may be precisely that of being able to improve cases of pneumonia management in limited-resource settings. To test this possibility, Lenahan et al. (2018), with their multicenter pilot study, proposed to pilot LUS in Mozambique and Pakistan and to generate evidence regarding the use of LUS as a diagnostic tool for childhood pneumonia.

In conclusion, the main purpose of all the studies was to evaluate whether LUS can be used as an alternative method to X-ray in the diagnosis of pneumonia. The consensus opinion of the aforementioned studies is that LUS has been proposed as a method with better sensitivity and specificity than CXR. The advantage of LUS is not only relative to the diagnosis of pneumonia, but also the lack of exposure to X-rays and the possibility of performing the examination at the patient’s bed as well as performing a follow-up.

However, some open questions did emerge. In particular, the question regarding how to determine when a negative LUS requires further evaluation with CXRs or whether it is safe not to prescribe antibiotics in cases of suspected pneumonia when LUS is normal or only shows interstitial syndrome or very small sonographic consolidations. Moreover, there is the need to standardize the appropriate protocol to interpret the LUS findings in childhood pneumonia and the need for international guidelines about the LUS use for pneumonia diagnosis. Future studies should focus on these aspects.

Future studies should also focus on ultrasound-based etiological diagnosis. Not only the presence or absence of the inflammatory event but the etiological hypothesis by which LUS could lead to a change in the current antibiotic therapy thereby encouraging personalized antibiotic treatment.

### Bronchiolitis

Bronchiolitis is a typical lower respiratory tract infection that usually affects children in the first two years of life. Its etiology is viral and more frequently it’s caused by Respiratory Syncytial Virus (RSV) (Meissner, 2016).

Bronchiolitis physiopathology is characterized by edema, increased production of mucus, and necrosis of cells of the small airway causing obstruction of distal bronchioles (Meissner, 2016).

In recent years, the clinical evaluation of children affected by bronchiolitis has been completed with LUS (Miller, 1995; Jaszczołt et al., 2018), exploiting the advantages of ultrasound especially in young children (non-invasive, non-ionizing radiation tool characterized by a rapid, affordable, point-of-care imaging modality that allows both real-time diagnosis and follow-up of respiratory diseases).

According to LUS findings (Ciaulo et al., 2011; Basile et al., 2015; Cohen et al., 2017; Di Mauro et al., 2019; Supino et al., 2019), the ultrasound pattern of bronchiolitis is not specified in an absolute sense. We can find it for example in the case of pneumonia of viral origin. It is characterized by the presence of a thickened pleura that reflects ultrasound waves from the sliding sign and by the presence of long vertical artifacts/B-lines and small subpleural consolidations. B-line/vertical artifacts derive from interstitial inflammation and/or disventilation. Inflammation in particular can be such as to also affect the alveolar component and therefore such as to cause the formation of a consolidation which can then become macroscopic and also characterized by elements of dynamic air bronchogram (Basile et al., 2015; Supino et al., 2019). Basile et al. (2015) proposed a US pattern classification that can be associated with the clinical classification. It has been described as having a good concordance between clinical condition and US pattern, highlighting a worse clinical course for those patients who have a subpleural consolidation with a diameter greater than 10 mm (Basile, 2015; Jaszczołt et al., 2018; Supino, 2019).

Lung ultrasound (LUS) shows better sensitivity than CXR in the detection of subpleural parenchymal consolidations of small dimensions (1–2 cm) (Jaszczołt et al., 2018) and it is also able to detect bacterial superinfection by detecting large consolidations and aerial bronchograms (Biagi et al., 2018).

In conclusion, considering the advantages of LUS over chest radiography in detecting and characterizing bronchiolitis findings and considering that there is a good concordance between clinical condition and ultrasonographic pattern (Ciaulo et al., 2011), LUS is especially important in the follow-up to avoid repeated chest radiographs. However, considering that the LUS findings of bronchiolitis are not specific, integration with clinical and laboratory data remains important in the diagnostic approach to the child with suspected bronchiolitis.

### Wheezing and Asthma

A field in which the use of LUS is not still well defined or is in development is that of asthmatic pathology. The studies available in the last ten years (from 2010 to 2020) in the literature are very few.

In the PED, children frequently present with respiratory distress and concomitant wheeze. Clinicians need to determine whether the pathophysiological process is one such as bronchiolitis, asthma, or pneumonia. The management of the aforementioned common conditions of childhood is dramatically different (Varshney et al., 2016; Dankoff et al., 2017).

A point-of-care tool that could differentiate between etiologies and/or guide the management of children with respiratory tract infections and wheeze would prove useful to the emergency care of these patients.

Varshney et al. (2016), performed the first study, a prospective study, featuring LUS findings in 94 children ≤2 years of age presenting to the PED with signs of a respiratory tract infection and wheeze. Among this category of children, a positive LUS seems to distinguish between clinical syndromes by ruling in pneumonia and ruling out asthma.

In 2017 same authors (Dankoff et al., 2017) performed the first study, a prospective study, characterizing lung ultrasound findings in children (aged between 2 and 17 years) with a moderate to severe acute asthma exacerbation. This study demonstrated that 45% of pediatric patients had a positive lung ultrasound during their acute respiratory presentation, of which 90% had a final physician diagnosis of asthma and 10% had asthma/pneumonia. Positive lung ultrasound was defined as the presence of ≥ 1 of the following findings: ≥ 3 B-lines per intercostal space, consolidation, and/or pleural abnormalities. Although the authors (Dankoff et al., 2017) have shown that pulmonary ultrasound is positive even in the course of asthma, they have not been able to define whether asthma, both in the acute phase and in the stability phase, is characterized by a specific LUS pattern.

Future prospective studies, better on larger pediatric populations, are needed to determine the usefulness and reliability of this tool in the clinical practice of asthma disease taking into account of (1) other diagnostic tests - and not just clinical evaluation associated with LUS evaluation - such as microbiological tests from the airways, chest X-ray, lung function assessment; (2) the treatment of acute asthma attack and any background therapy performed; (3) changes in the LUS findings and other clinical and instrumental assessments before and after the administration of therapy for acute attack; (4) characterization of the ultrasound lung pattern in patients with stable asthma and its comparison with other instrumental and functional evaluations.

### Lung Ultrasound in Other Specific Settings

#### Lung Ultrasound After Cardiac Surgery

The performance of LUS approaches that of chest CT and surpasses that of CXR for diagnosing lung diseases that occur frequently after cardiac surgery, including consolidation, pleural effusion, pulmonary edema, and pneumothorax (Ashton-Cleary, 2013). Song et al. (2018) published a randomized controlled trial with the aim of assessing the utility of perioperative LUS and the effect of US-guided recruitment maneuver in pediatric cardiac surgery taking into account that the optimization of perioperative respiratory care is crucial for improving outcomes after pediatric cardiac surgery. Lung ultrasound findings (degree of consolidations, B-lines, and pleural effusion) were characterized and evaluated following the evaluation method described by Song et al. (2017). According to the authors, perioperative LUS examination followed by ultrasound-guided recruitment maneuver helped decrease postoperative desaturation events and shortened the duration of mechanical ventilation in pediatric cardiac patients.

In particular, among 120 children included in the analysis (aged 5 years or less and divided into 60 in the control group and 60 in the intervention group), the postoperative desaturation occurred more in the control group. LUS scores were better in the intervention group than in the control one. Duration of mechanical ventilation was longer in the control group than in the intervention group. In this way, the authors encouraged more active LUS application in pediatric cardiac surgery (Song et al., 2018).

Also, according to Tripathi et al. (2019), who performed a prospective observational study at PICU using the ultrasound image acquisition protocol in the critically ill (Lichtenstein, 2014), LUS provides actionable quantitative data and it’s useful to monitor lung recruitment and other dynamic changes.

#### Lung Ultrasound in Cystic Fibrosis

The aim of the pilot study of Strzelczuk-Judka et al. (2019) was to evaluate the diagnostic value of LUS in children with *Cystic Fibrosis (CF)* compared to a CXR scoring system and to assess the diagnostic value of the recently developed LUS score CF-USS (Cystic Fibrosis Ultrasound Score), devised based on the modified Chrispin Norman score and the bronchiolitis score reported by Caiulo and colleagues who applied LUS in patients with bronchiolitis, which is also present in CF patients (Brasfield et al., 1980; Helbich et al., 1999; Caiulo et al., 2011, 2013; Strzelczuk-Judka et al., 2019). In each patient, the authors evaluated: – the quality and quantity of any fluid in the pleural space; - the shape and thickness of the pleural line, the lung sliding sign; – A-lines and B-lines artifacts (number, localization, and morphology, including single ones, “lung rockets” complexes and “white lung” images) and – the alveolar consolidations (number, dimensions, localization, morphology, presence of bronchogram and its air or fluid characteristic and vascularization). Lung ultrasound findings were also classified according to CF-USS. According to the authors, LUS should be a supplementary examination in scheduled follow-up visits in pediatric patients with CF, and the CF-USS scoring system could provide clinicians with valuable information on disease progression. The CF-USS results correlated with the conventional x-ray modified Chrispin–Norman score. Moreover, the authors emphasize that LUS could constitute an invaluable tool for the diagnosis of subpleural consolidations (Strzelczuk-Judka et al., 2019).

However, according to the authors of the studies described above (Song et al., 2018; Strzelczuk-Judka et al., 2019; Tripathi et al., 2019), LUS has low negative predictive values and negative examinations and cannot rule out lung pathology. Limitations of LUS would include the inability to visualize consolidations separated from the pleura and larger airways. The numerous clinical conditions in which B-line artifacts could be present also make it difficult to recommend LUS as the only diagnostic modality in some categories of patients such as those with underlying lung diseases (e.g., CF) (Song et al., 2018; Strzelczuk-Judka et al., 2019; Tripathi et al., 2019).

## Disadvantages of Lung Ultrasound

Many studies have commented on the disadvantages of LUS. These disadvantages included the following (Copetti, 2016; Lovrenski et al., 2016; Cox et al., 2017; Lovrenski, 2020): (1) the inability to visualize the paravertebral regions (beneath the scapulae); (2) the difficulty in examining some patients who are characterized as hypoechogenic (e.g., patients with obesity, subcutaneous emphysema, dressings/wounds); (3) the non-optimal position in which patients with acute respiratory distress and dyspnea are often found, which limits the examination of some lung areas; (4) the air leak syndromes (pneumomediastinum, pneumopericardium, and interstitial emphysema) which couldn’t be easily identified by LUS; (5) the areas of air trapping and hypoalveolarization that characterize much of the child’s chronic pathology; (6) the errors that may occur if the operator is not properly trained and experienced.

Furthermore, in the context of infectious pulmonary disease, specifically pneumonia, LUS findings are considered non-specific from an etiological point of view and need to be compared and associated with clinical and laboratory findings in order to determine the true etiology of pulmonary changes. No studies have yet investigated the role of LUS in the etiological diagnosis of pediatric pneumonia.

## What Are the Future Diagnostic Challenges of Lung Ultrasound?

Lung ultrasound (LUS) in pediatrics could be used to expand and improve its diagnostic capabilities in several ways. These would include the better capability in the following aspects. First, in the differential diagnosis between viral and bacterial lower respiratory infections and diagnosis of interstitial pneumonia in which the application of LUS could determine the etiological diagnosis of pneumonia in children which could improve antibiotic stewardship (Berant et al., 2015; Han et al., 2018). Second, in the qualitative, quantitative, and objective characterization of vertical artifacts in each of the childhood respiratory diseases, which include chronic disabling ones, through the development of artificial intelligence to identify more specific ultrasound patterns and to create ultrasound scores as is going on for lung infection by COVID-19 (Soldati et al., 2020; Vetrugno et al., 2020). Third, in the use of contrast-enhanced lung ultrasound in complicated pneumonia, both intravenous and intracavitary: intravenous contrast-enhanced US can accurately diagnose “necrotizing” pneumonia and delineate pleural effusion, while intracavitary contrast-enhanced US can identify the location and patency of the thoracic catheter and show the presence of loculations (Lovrenski, 2020). Fourth, in the correlation of LUS with clinical parameters, mechanical ventilation parameters in the context of neonatal and pediatric intensive care; LUS also has the potential for enabling a more focused pulmonary rehabilitation of these children after extubating, targeting precisely to the poorly ventilated areas that need treatment. Finally, in the correlation between LUS and CT findings by performing LUS after each chest CT, which is the gold standard of lung diseases, which could be indicated for other reasons.

## Conclusion

The greatest potential of LUS belongs to the dynamic follow-up of pulmonary conditions, making many everyday decisions easier for clinicians and enabling a higher quality of treatment and faster recovery of children. For some conditions, such as lung consolidations of both infectious and non-infectious nature, LUS can be considered the first-choice tool for the diagnosis and follow-up of pediatric lung diseases.

Conversely, whenever there is a discrepancy between LUS and other findings (clinical and laboratory), CXR or CT (in more complicated cases) should be performed. This kind of approach would probably reduce the number of CXRs significantly.

Importantly, one of the biggest advantages of LUS is its availability, especially in developing countries (Buonsenso and De Rose, 2021), because it is often easier to bring the ultrasound device to children than to conduct the children to ultrasound rooms of distant hospitals. Unlike most US techniques, LUS does not require a high-tech ultrasound device: old ultrasound devices and two US probes (convex and linear) are sufficient to provide the necessary information. Fortunately, LUS techniques are easy to perform and master so they are very convenient both outside and inside hospitals; however, as in any other aspect of life, experience is important for lung US, as well (Lovrenski, 2020). The best results are obtained by choosing case by case by integrating the different diagnostic tools.

In conclusion, over the past 10 years, LUS has enhanced our ability to diagnose many pediatric respiratory conditions. Further studies are ongoing that will help us to integrate LUS with the other more commonly used diagnostic modalities to an even greater extent.

## Author Contributions

All authors have read and approved the manuscript for submission, and have made a substantial contribution to the conception, design, gathering of data, and contribution to the writing and intellectual content of the article.

## Conflict of Interest

The authors declare that the research was conducted in the absence of any commercial or financial relationships that could be construed as a potential conflict of interest.

## Publisher’s Note

All claims expressed in this article are solely those of the authors and do not necessarily represent those of their affiliated organizations, or those of the publisher, the editors and the reviewers. Any product that may be evaluated in this article, or claim that may be made by its manufacturer, is not guaranteed or endorsed by the publisher.
